# 

**Published:** 1880-07

**Authors:** 


Vol. I.] TWO DOLLARS A YEAR. SINGLE COPIES 50 CENTS. [No. 3.

### THE ARCHIVES

OF

### COMPARATIVE MEDICINE AND SURGERY.

### A Quarterly Journal

OF THE

### ANATOMY, PATHOLOGY, AND THERAPEUTICS OF THE LOWER ANIMALS.

NEW YORK:
W. L. HYDE & CO., PUBLISHERS, NO. 22 UNION SQUARE.
1880.

